# CRISPR-Mediated *Slamf1^Δ/Δ^ Slamf5^Δ/Δ^ Slamf6^Δ/Δ^* Triple Gene Disruption Reveals NKT Cell Defects but Not T Follicular Helper Cell Defects

**DOI:** 10.1371/journal.pone.0156074

**Published:** 2016-05-25

**Authors:** Joyce K. Hu, Jordan C. Crampton, Michela Locci, Shane Crotty

**Affiliations:** 1 Division of Vaccine Discovery, La Jolla Institute for Allergy and Immunology, La Jolla, California, United States of America; 2 Department of Medicine, University of California, San Diego School of Medicine, La Jolla, California, United States of America; Jackson Laboratory, UNITED STATES

## Abstract

SAP (SH2D1A) is required intrinsically in CD4 T cells to generate germinal center responses and long-term humoral immunity. SAP binds to SLAM family receptors, including SLAM, CD84, and Ly108 to enhance cytokine secretion and sustained T cell:B cell adhesion, which both improve T follicular helper (Tfh) cell aid to germinal center (GC) B cells. To understand the overlapping roles of multiple SLAM family receptors in germinal center responses, *Slamf1*^Δ/Δ^ *Slamf5*^Δ/Δ^ *Slamf6*^Δ/Δ^ triple gene disruption (*Slamf1*,*5*,*6*^Δ/Δ^) mice were generated using CRISPR-Cas9 gene editing to eliminate expression of SLAM (CD150), CD84, and Ly108, respectively. Gene targeting was highly efficient, with 6 of 6 alleles disrupted in 14 of 23 pups and the majority of alleles disrupted in the remaining pups. NKT cell differentiation in *Slamf1*,*5*,*6*^*Δ/Δ*^ mice was defective, but not completely absent. The remaining NKT cells exhibited substantially increased 2B4 (SLAMF4) expression. Surprisingly, there were no overt defects in germinal center responses to acute viral infections or protein immunizations in *Slamf1*,*5*,*6*^Δ/Δ^ mice, unlike *Sh2d1a*^-/-^ mice. Similarly, in the context of a competitive environment, SLAM family receptor expressing GC Tfh cell, GC B cell, and plasma cell responses exhibited no advantages over *Slamf1*,*5*,*6*^Δ/Δ^ cells.

## Introduction

Germinal center (GC) responses are critical for the generation of high affinity antibodies (Abs), memory B cells, and long-lived plasma cells. T follicular helper (Tfh) cells are subset of CD4 T cells specialized in supporting and regulating GC responses [1,2]. Tfh cells provide help signals that support GC B cell survival, proliferation, and somatic hypermutation and promote plasma cell differentiation via receptors like CD40L and through secreted factors like IL-21 [3,4]. These cognate interactions between Tfh cells and GC B cells are regulated by SLAM family receptors and SLAM-associated protein (SAP, *Sh2d1a*). Therefore, understanding how these receptors modulate Tfh cells and regulate GCs can be useful for developing rational vaccine immunology as well as potential therapeutics for autoantibody-associated autoimmune diseases.

Defects in *Sh2d1a* in humans causes X-linked lymphoproliferative disease (XLP), a disorder with high mortality characterized by the inability to clear infections and dysregulated T cell responses [5]. SAP deficient humans and mice have impaired generation of germinal centers (GCs), isotype-switched memory B cells, and circulating antibodies [6–10]. These defects can be rescued in mice via transfer of SAP sufficient CD4 T cells, demonstrating the critical cell intrinsic role of SAP in CD4 T cells [7,11]. Therefore, understanding the role of SAP in CD4 T cells is critical to the mechanistic understanding of defective humoral responses in XLP patients.

SAP binds to SLAM family (SLAMf) receptors, a family of nine receptors selectively expressed on cell types of the hematopoietic lineage. CD4 T cells express the SAP-binding SLAMf receptors SLAM (SLAMF1), Ly9, CD84 (SLAMF5), and Ly108 (SLAMF6) [5] and these receptors regulate different T cell functions. All four receptors are homophilic ligands. Single SLAMf KO mice have modest, if any, defects in the magnitude of Tfh or GC responses [12–15], in stark contrast to the severe defects observed in SAP-deficient animals. *Slamf1*^-/-^ Tfh cells have significantly reduced IL-4 expression [15,16], but the overall magnitude of the Tfh and GC responses are unchanged. The generation of pseudo double knockout conditions was useful to examine phenotypes involving interacting cells and SLAMf redundancies. For example, examining interactions between CD84 KO CD4 T cells with Ly108 KO B cells led to the finding that CD84 and Ly108 both contributed to sustained T cell:B cell adhesion [13]. Similarly, it was shown that SLAM and Ly108 were the primary receptors involved in NKT cell development by using Ly108 KO NKT cell precursors with SLAM KO thymocytes expressing CD1d [17]. Likewise, there may be additional roles that SLAMf receptors play that are unknown due to redundancy. Furthermore, Ly108 transmits both positive and negative signals [12,18,19], additionally confounding the interpretation of single SLAMf KO mice. *Slamf6*^*-/-*^*Sh2d1a*^*-/-*^ mice exhibit substantially rescued GC Tfh cells and germinal center responses, demonstrating that Ly108 transmits potent negative signals in the absence of SAP. Ly108 transmits positive signals in NKT cells [12], NK cells [20], and CD8 T cells [18,19], but this was not directly observable in CD4 T cells. Thus, generating multi-SLAMf receptor gene deficient mice is a useful way to gain a more comprehensive understanding of SLAMf receptor function. However, because the SLAMf genes are located adjacent to each other on chromosome 1 in a large cluster, it has been very challenging to make multi-SLAMf receptor knockouts and this has hindered research in this area. A *Slamf1*^*-/-*^*Slamf5*^*-/-*^*Slamf6*^*-/-*^ (*Slamf1*,*5*,*6*^*-/-*^) mouse has recently been reported [21,22]; however that genetic modification involved deletion of 140,000 bp of the genome, which could have unpredictable effects on gene regulation of the many neighboring SLAMf genes. Therefore, we employed CRISPR-Cas9 gene editing strategies to generate multi-gene deficient mice to examine the roles of multiple SLAM family receptors.

## Materials and Methods

### Ethics statement

The mouse experiments were conducted in compliance with the La Jolla Institute for Allergy and Immunology Animal Care Committee (Office of Laboratory Animal Welfare assurance number A3779-01), who approved all animal care and protocols used (Protocol license number AP006-SC1-0612). The mouse care and use protocol adheres to the Public Health Service (PHS) Policy on the Humane Care and Use of Laboratory Animals (Department of Health and Human Services) and the Guide for the Care and Use of Laboratory Animals (eighth edition). Mice were anesthetized with isoflurane and euthanized with a carbon dioxide gas chamber followed by cervical dislocation.

### Generation of SLAM^Δ/Δ^, CD84^Δ/Δ^, and Ly108^Δ/Δ^ mice using CRISPR-Cas9 gene editing technology

The *in vitro* molecular and cellular biology was performed by Applied Stem Cell, Inc. Guide RNAs were selected using optimized CRISPR design by the Feng Zhang lab (crispr.mit.edu). Guide RNAs were further selected based on the criteria that they target the second exon of each receptor, target all isoforms of each receptor, and be unique for the targeted sites with up to two base pair mismatches. Also, 5’G motifs [23] and 3’ purines were preferred [24]. Oligos for each of the gRNAs were cloned into the gRNA expression vector pBT-U6-Cas9-2A-GFP (or pX330 hSpCas9 vector with 2a-EGFP from the Feng Zhang lab). To test the activity of each gRNA, the gRNA expressing vectors were transfected into mouse N2A cells and the Surveyor assay was performed according to the manufacturer’s instructions. Linearized pBT-T7-Cas9 plasmid was used as the template for *in vitro* transcription (IVT) using mMESSAGE mMACHINE T7 ULTRA kit (Life Technologies). T7 promoter was added to each gRNA template by PCR, gel purified, and used as a template for IVT using MEGAshortscript T7 kit (Life Technologies). Cas9 mRNA and gRNAs were purified using MEGAclear kit (Life Technologies) and eluted in RNA elution buffer. To test the activity of Cas9 mRNA, Cas9 mRNA was translated into protein using 1-Step Human IVT kit (Thermo Scientific) per instructions. An *in vitro* cleavage assay showed >95% IVT Cas-9 activity. An injection mix of 50 ng/μl Cas9 mRNA, 50 ng/μl SLAM-gRNA, 50 ng/μl CD84-gRNA, and 50 ng/μl Ly108-gRNA was injected into 150–250 one-cell embryos from C57BL/6J mice by the UCSD Stem Cell Core. These embryos were implanted into C57BL/6J surrogate mothers, and pups were genotyped by DNA sequencing and phenotyping by flow cytometry. DNA sequences were analyzed using Sequencher and diagrammed using SnapGene.

### Mice, infections, and immunizations

Six to eleven week old age-matched wild-type (WT) or SLAM^Δ/Δ^ CD84^Δ/Δ^ Ly108^Δ/Δ^ mice (on a C57BL/6J background) were infected intraperitoneally with 2x10^5^ plaque forming units (PFU) of lymphocytic choriomeningitis virus (LCMV; Armstrong strain), intraperitoneally with 2x10^6^ PFU Vaccinia virus (VACV; Western Reserve strain), or via footpads with 20 μg HIV envelope trimer protein (YU2 gp140-Foldon) in Addavax adjuvant (Invivogen). Bone marrow chimeras were generated by treating 6–8 week old WT SJL-Ptprc^a^ Pepc^b^/BoyJ (B6.SJL) recipient mice with antibiotics (Equisul) for 3–5 days, irradiating mice with 2 doses of 500 rads from a Cesium source a few hours apart, and on the same day, injecting 1x10^6^ CD45.1 WT and either 1 x 10^6^ CD45.2 WT or 1x10^6^ CD45.2 *SLAMf1*,*5*,*6*^Δ/Δ^ bone marrow cells from age-matched 6–8 week old mice. Mice were reconstituted with bone marrow cells for 8–10 weeks before infection with 2x10^5^ PFU LCMV-Armstrong or 2x10^6^ PFU VACV-WR. For NKT cell phenotypic analysis, livers and spleens were collected, processed, and stained as previously reported [12]. For NKT cell functional assays, 1 ug of a-GalCer was injected intravenously and mice were euthanized 45 minutes later to assess the *ex vivo* production of IL-4 and IFN-γ by NKT cells.

### Flow cytometry

Stains were performed as previously described [25]. SLAM family receptor expression levels were measured using anti-mouse SLAM (Biolegend; TC15-12F12.2), anti-mouse CD84 (Biolegend; mCD84.7), and anti-mouse Ly108 (eBioscience; eBio13G3-19D).

For Bcl6 staining, cells were stained for surface markers, then fixed and permeabilized with fixation and permeabilization buffer (eBioscience) per manufacturer’s protocol, and stained with anti-Bcl6 monoclonal antibody (K112-91, BD Biosciences). For CXCR5 staining, cells were stained with purified rat anti-mouse CXCR5 (2G8, BD Biosciences) in PBS + 0.5% BSA + 2% normal mouse serum + 2% FCS (FACS buffer) for 1 hour, followed by goat anti-rat (H+L, Jackson Immunoresearch) in FACS buffer for 30 minutes, followed by other surface stains with washes in between each step.

### Enzyme-linked immunosorbent assay (ELISA)

ELISAs were performed as previously described [26]. Briefly, Maxisorp plates were coated with Vaccinia virus antigen or HIV envelope trimers (YU2-gp140-F) overnight at 4°C. The plates were blocked with PBS + 0.1% Tween 20 + 0.5% BSA, mouse serum was added to plates, and HRP-labelled anti-mouse IgG (fragment specific Fcγ) was added with washing steps using PBS + 0.1% Tween 20 in between each step. Colorimetric development was performed with the TMB substrate kit and stopped with H_2_SO_4_, followed by measurement of absorption at 450 nm. Analysis was performed to measure endpoint titers (0.1 OD above background) and Area under curve (AUC). AUC analysis better accounts for both the quantity and quality of the IgG, as it accounts for the shape of the curve. AUC total peak area above baseline calculations (Graphpad Prism 6.0) were done for each individual sample, log transformed.

### Statistics

Prism 6 Software was used to plot geometric mean and geometric standard error of the mean for log-based graphs, or mean and standard error of the mean for linear-based graphs. Statistical analysis was performed using Mann-Whitney t-tests.

## Results

### Generation of *Slamf1*^Δ/Δ^ *Slamf5*^Δ/Δ^ *Slamf6*^Δ/Δ^ triple gene disruption (*Slamf1*,*5*,*6*^Δ/Δ^) mice using CRISPR-Cas9 technology

**Fig 1:**
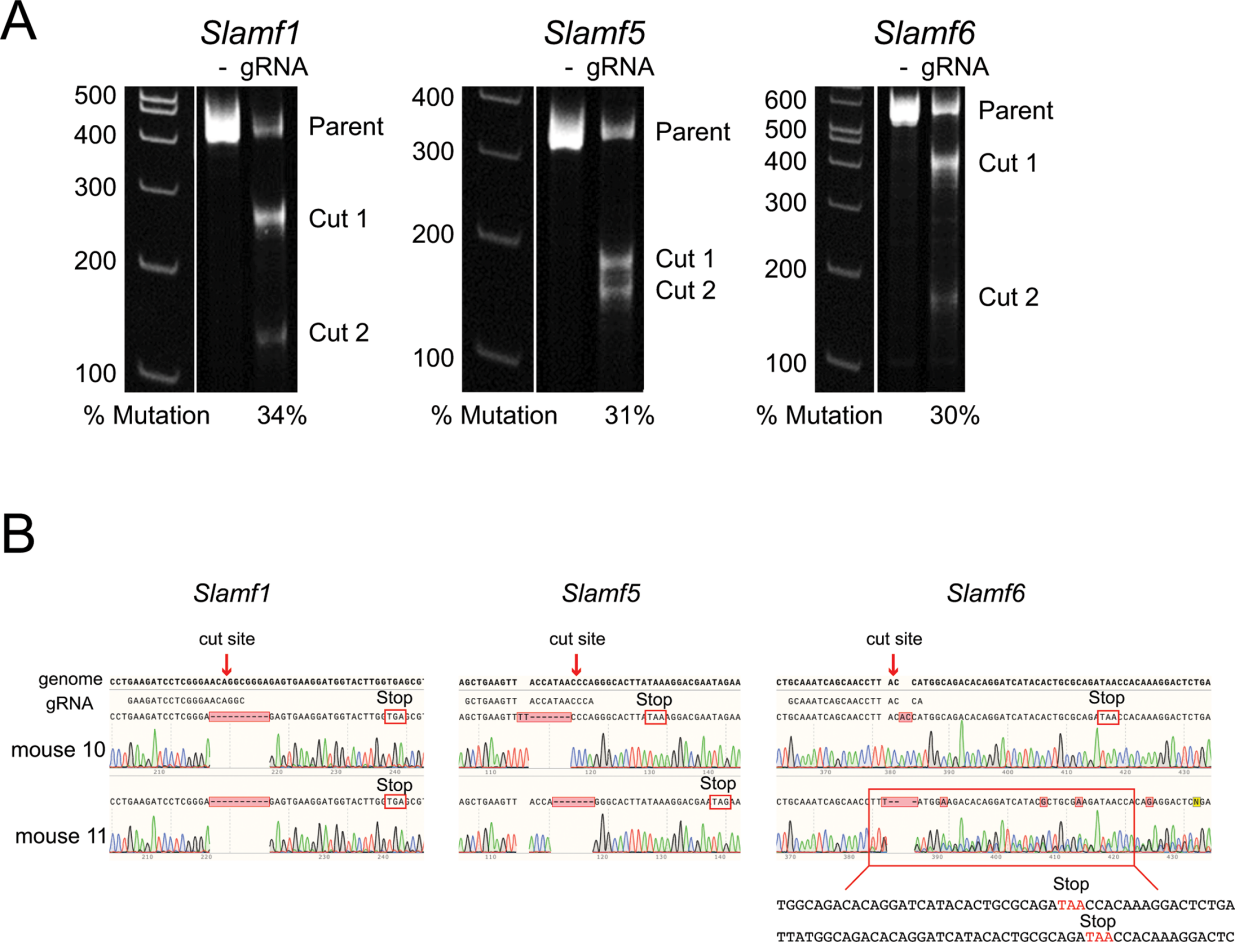
Generation of *Slamf1*,*5*,*6* ^Δ/Δ^ mice using CRISPR-Cas9 technology. (A) Surveyor assay performed to determine mutation efficiency in mouse N2A cells that were transfected with gRNA expressing pBT-U6-Cas9-2A-GFP vectors. Gel shows comparison between the original gene product (parent) and the mutated gene products (cut 1, cut 2). One surveyor assay was performed. (B) Genotyping of *Slamf1*^Δ/Δ^, *Slamf5*^Δ/Δ^, *Slamf6*^Δ/Δ^ mice. Genomic DNA, guide RNA (gRNA), and sequencing results are aligned, with shaded boxes showing differences in sequence, which include deletions (dashes), insertions, and uncertain base calling that results from heterozygous mutations in each allele. The sequences of each allele of *Slamf6* in mouse 11 is shown beneath the chromatogram. Each mutation leads to a stop codon shown in a red box. Genotyping was performed as indicated in Table 2.

To determine the role of multiple SLAM family (SLAMf) receptors in GC responses, *Slamf1*^Δ/Δ^ *Slamf5*^Δ/Δ^ *Slamf6*^Δ/Δ^ triple gene disruption (*Slamf1*,*5*,*6*^Δ/Δ^) mice were generated. Given the sequential location of these genes at the same gene locus, the rare occurrence of chromosomal crossover between them rendered it unfeasible to create triple knockout (TKO) mice by crossing single SLAMf receptor single knockout (KO) mice. Thus, CRISPR-Cas9 gene-editing technology was employed to knockout all three receptors. CRISPR-Cas9 uses guide RNAs (gRNAs) and the Cas9 DNA endonuclease to cut specific sequences in the genome [24,27–31]. Unique 20 base pair sequences were designed to target each SLAMf receptor and gRNA sequences were chosen that had no off-target gene matches with up to two base pair mismatches (Table 1). The efficiency of gene targeting using these gRNAs was 30–34 percent as quantified *in vitro* via Surveyor assay (Fig 1A). One-cell embryos were then injected with *Slamf1*, *Slamf5*, and *Slamf6* gRNAs and Cas9 mRNA and embryos were implanted into surrogate mothers. This resulted in 23 viable pups, which were sequenced for mutations in *Slamf1*, *Slamf5*, and *Slamf6* and/or phenotyped for the expression of the receptors SLAM, CD84, and Ly108 (Table 2). Error-prone DNA repair resulted in insertions, deletions, or a combination of the two, at the targeted cut site and this led to frameshifts and stop codons (Fig 1B). Strikingly, null mutations were generated in all 6 gene alleles for 14 of 23 pups. The remaining 9 pups were then partially characterized, with 8 of 9 confirmed to be at least double knockout (DKO). We then established three separate breeder lines (Table 2; breeders: #4 and #21, #11 and #23, #14 and #22), which each gave identical immunological results (S1 Fig). In sum, CRISPR-Cas9 targeting of SLAM family genes in embryos was highly efficient.

**Table 1:** gRNA sequences and characteristics.

Gene	gRNA sequence	Mutation efficiency	Off-target genes		
			0 mismatches	1 mismatch	2 mismatches
*Slamf1* (SLAM)	GAAGATCCTCGGGAACAGGC	34%	0 genes	0 genes	0 genes
*Slamf5* (CD84)	GCTGAAGTTACCATAACCCA	31%	0 genes	0 genes	0 genes
*Slamf6* (Ly108)	GCAAATCAGCAACCTTACCA	30%	0 genes	0 genes	0 genes

Table of guide RNA (gRNA) sequences used for each gene, the mutation efficiency seen by expression of each gRNA in mouse N2A cells, and the number of predicted off-target sites in the genome using each gRNA sequence with up to two base pair mismatches.

**Table 2:** Genotyping and phenotyping of CRISPR gene edited mice.

No.	Gender	Genotyping			Phenotyping			Result
		*Slamf1* (SLAM)	*Slamf5* (CD84)	*Slamf6* (Ly108)	SLAM	CD84	Ly108	
1	Male	Hom (stop, exon2)	Het (stop, exon2; no stop)	Het (stop, exon2)	n.d.	n.d.	n.d.	Tmut*
2	Male	n.d.	n.d.	Het (stop, exon2)	NULL	NULL	NULL	Tnull
3	Male	Hom (stop, exon2)	n.d.	n.d.	n.d.	NULL	NULL	Tnull
4	Male	Hom (stop, exon2)	Het (stop, exon2)	Het (stop, exon2)	n.d.	n.d.	n.d.	Tnull
5	Male	n.d.	Hom (stop, exon2)	n.d.	NULL	NULL	NULL	Tnull
6	Male	n.d.	Hom (stop, exon 2)	Het (stop, exon2)	NULL	NULL	NULL	Tnull
7	Male	Hom (stop, exon2)	n.d.	Hom (stop, exon2)	NULL	NULL	NULL	Tnull
8	Male	n.d.	n.d.	n.d.	NULL	n.d.	NULL	Dnull*
9	Male	n.d.	n.d.	Hom (stop, exon2)	NULL	n.d.	NULL	Dnull*
10	Male	Hom (stop, exon2)	Hom (no stop)	Hom (stop, exon2)	n.d.	n.d.	n.d.	Tmut*
11	Male	Hom (stop, exon2)	Hom (stop, exon2)	Het (stop, exon2)	n.d.	n.d.	n.d.	Tnull
12	Male	n.d.	Hom (stop, exon2)	n.d.	NULL	n.d.	NULL	Tnull
13	Male	Hom (stop, exon2)	n.d.	Hom (stop, exon2)	n.d.	n.d.	n.d.	Dnull*
14	Male	Hom (stop, exon3)	Het (stop, exon2)	Hom (stop, exon3)	n.d.	n.d.	n.d.	Tnull
15	Male	n.d.	n.d.	n.d.	n.d.	n.d.	NULL	n.d.
16	Male	Hom (stop, exon2)	n.d.	n.d.	n.d.	n.d.	NULL	Dnull*
17	Female	n.d.	n.d.	n.d.	NULL	n.d.	NULL	Dnull*
18	Female	n.d.	Hom (stop, exon3)	Het (stop, exon3)	NULL	n.d.	NULL	Tnull
19	Female	n.d.	Hom (no stop)	Hom (stop, exon2)	NULL	n.d.	n.d.	Tmut*
20	Female	n.d.	n.d.	n.d.	NULL	n.d.	NULL	Dnull*
21	Female	Hom (stop, exon2)	Het (stop, exon2)	n.d.	n.d.	n.d.	NULL	Tnull
22	Female	Hom (stop, exon2)	Het (stop, exon2)	n.d.	n.d.	n.d.	NULL	Tnull
23	Female	Hom (stop, exon2)	Hom (stop, exon2)	n.d.	n.d.	n.d.	NULL	Tnull

Table of mouse genotyping and phenotyping showing a high efficiency of genetic mutation resulting in mice deficient in the expression of multiple SLAM family receptors.

Hom, homozygous mutation; Het, heterozygous mutation; Stop, stop codon (TAA, TAG, or TGA) induced by gene editing; n.d., not determined; Tnull, triple gene mutations inducing null expression; Tmut*, triple gene mutations that did not result in null expression; Dnull*, double gene mutations inducing null expression, editing of third gene not determined; NULL, null protein expression as determined by surface stain.

### *Slamf1*^Δ/Δ^ *Slamf5*^Δ/Δ^ *Slamf6*^Δ/Δ^ triple gene disruption mice generated similar frequencies of peripheral T cells and B cells but decreased frequencies of NKT cells

**Fig 2:**
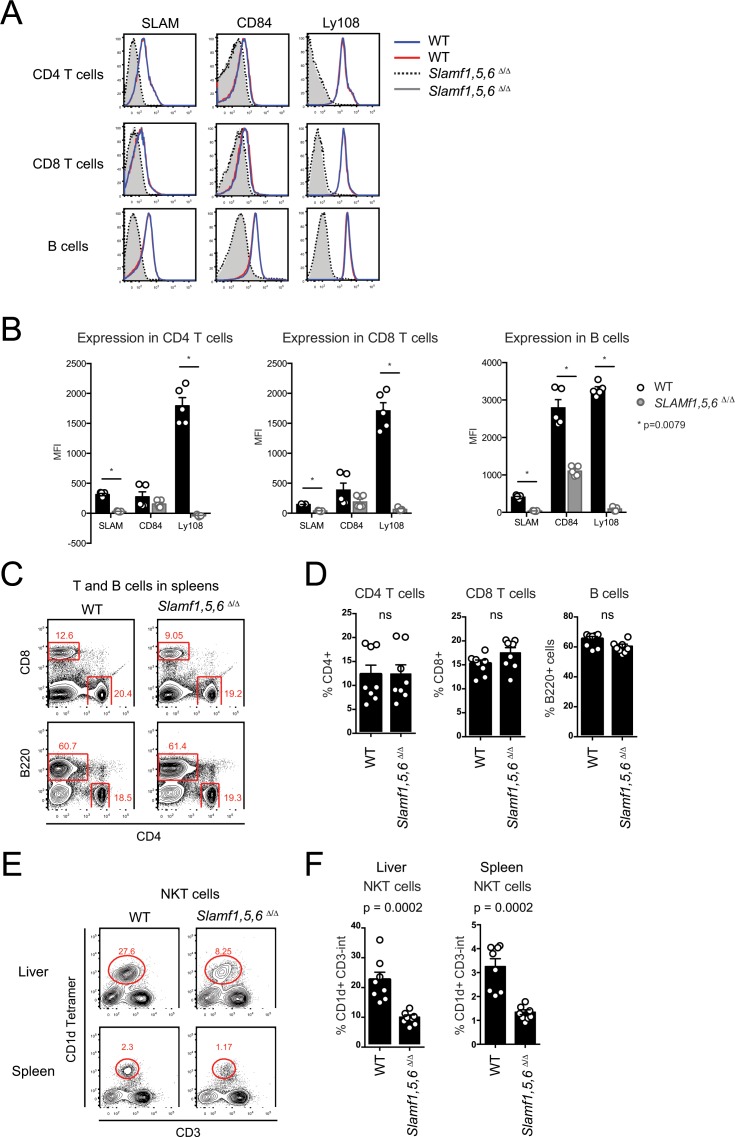
*Slamf1*,*5*,*6* ^Δ/Δ^ mice exhibit deficiencies in NKT cell development but no overt defects in CD4 T cell, CD8 T cell, or B cell development. (A) Surface expression and (B) MFI of SLAM, CD84, and Ly108 on peripheral CD4 T cells, CD8 T cells, and B cells from WT and SLAMf receptor triple gene disruption mice. (A-B) Two independent experiments are shown, with 2–3 mice per group. (C) Flow cytometry plots and (D) graphs of CD4^+^ T cell, CD8^+^ T cell, and B220^+^ B cell frequencies in spleens of WT and SLAMf receptor triple gene disruption mice. (C-D) Data is representative of two independent experiments, with 4 mice per group. (E) Flow cytometry plots and (F) graphs of B220^-^ CD3^+^ CD1d Tetramer^+^ NKT cells in spleens and livers of WT and SLAMf receptor triple gene disruption mice. (E-F) Data are representative of 2 independent experiments, with 4 mice per group.

To determine if the SLAMf receptors impacted the generation of peripheral lymphocytes in *Slamf1*,*5*,*6*^Δ/Δ^ mice, peripheral CD4 T cells, CD8 T cells, B cells, and NKT cells were quantified. Surface expression of SLAM, CD84, and Ly108 was absent on CD4 T cells, CD8 T cells, and B cells in *Slamf1*,*5*,*6*^Δ/Δ^ mice compared to wild-type (WT) mice (Fig 2A and 2B). Residual signal by the anti-CD84 mAb binding B cells suggests a crossreactivity of the mAb for another antigen on B cells, as no signal was apparent on CD4 T cells or CD8 T cells, and homozygous *Slamf5* stop mutations were confirmed in the genome (S2 Fig). Frequencies of peripheral CD4 T cells, CD8 T cells, and B cells were similar (Fig 2C and 2D), suggesting that SLAM, CD84, and Ly108 are not required for the development of peripheral T and B cells. In contrast, peripheral NKT cells were reduced approximately 3-fold in liver and 2.5-fold in spleen of *Slamf1*,*5*,*6*^Δ/Δ^ mice compared to WT (p = 0.0002) (Fig 2E and 2F). Surprisingly, this NKT phenotype was less severe than that seen in SAP-deficient mice [32,33] and moderately more severe than *Slamf6*^-/-^ (Ly108-deficient) mice [12,17]. Previously, it was thought that SLAM and Ly108 were the primary SLAMf receptors involved in NKT cell development [17]. The data here, and from the *Slamf1*,*5*,*6*^-/-^ mice [21], suggest that additional SLAMf receptors, or SAP binding receptors other than SLAM, CD84, and Ly108, contribute to NKT cell development.

**Fig 3:**
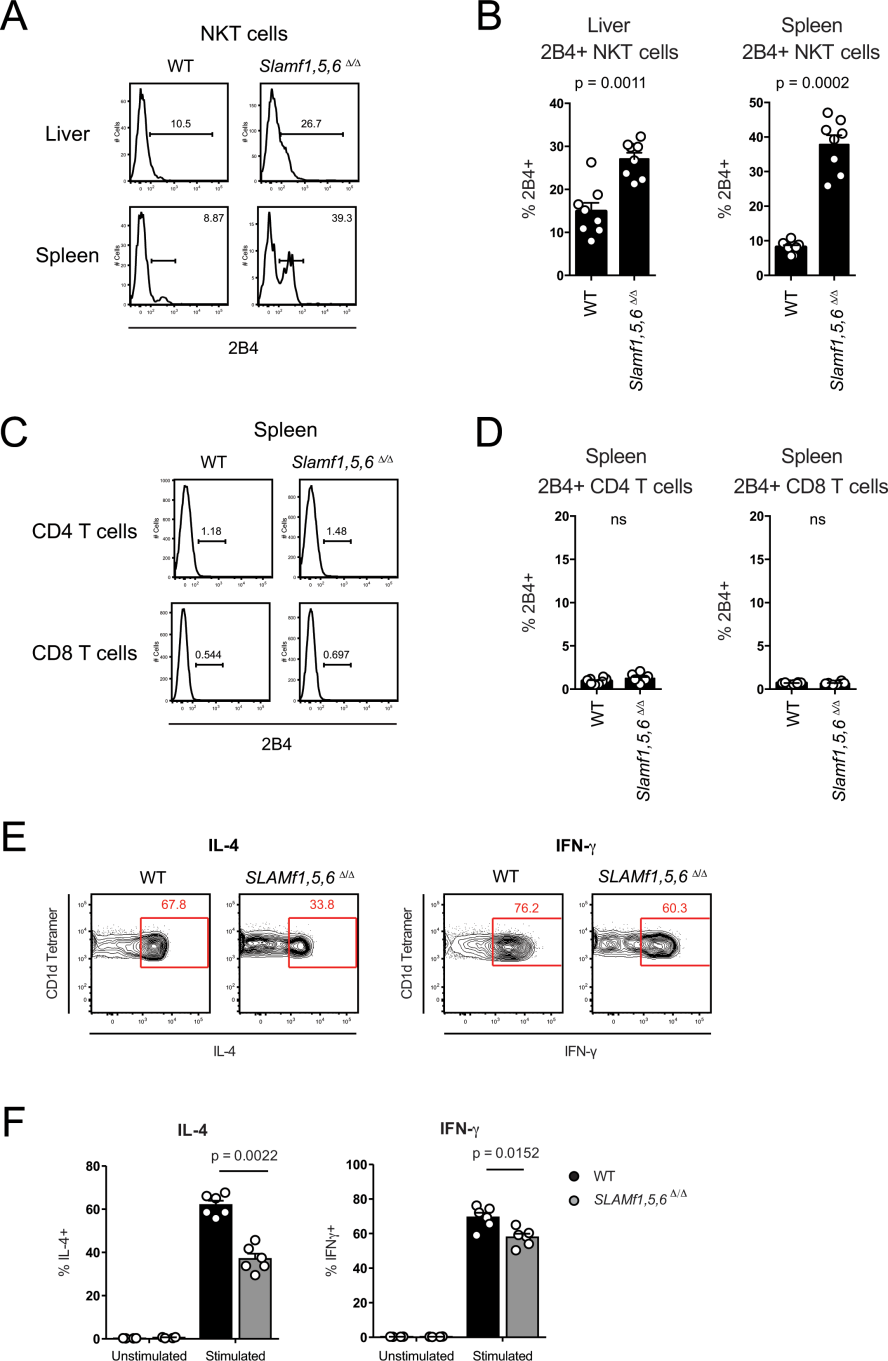
NKT cells from *Slamf1*,*5*,*6* ^Δ/Δ^ mice have higher levels of 2B4 expression and lower functional secretion of cytokines. (A) Histograms and (B) graphs of 2B4 surface expression on B220^-^ CD3^+^ CD1d Tetramer^+^ NKT cells in spleens and livers of WT and *Slamf1*,*5*,*6* ^Δ/Δ^ mice. (C) Histograms and (D) graphs of 2B4 surface expression on splenic CD4 and CD8 T cells in WT and *Slamf1*,*5*,*6* ^Δ/Δ^ mice. (A-D) Data shows two independent experiments, with 4 mice per group. (E) Flow cytometry plots of representative WT and *Slamf1*,*5*,*6* ^Δ/Δ^ NKT cells (gated on B220^-^ CD3^int^ CD1d Tetramer^+^ cells) after *in vivo* stimulation with α-GalCer for 45 minutes. Expression of IL-4 and IFN-γ are shown. (F) Frequencies of IL-4 and IFN-γ expression by NKT cells (gated on B220^-^ CD3^int^ CD1d Tetramer^+^ cells). (E-F) Data represents two independent experiments, with 3 mice per group.

Given that developing NKT cells can express other SLAMf receptors [34], there may be roles for these receptors in NKT cell development. To examine this possibility, we investigated 2B4 (CD244, SLAMF4) expression. Interestingly, *Slamf1*,*5*,*6*^Δ/Δ^ mice have much larger populations of 2B4^+^ NKT cells in both livers (p = 0.0011) and spleens (p = 0.0002) (Fig 3A and 3B). Expression of 2B4 on naïve CD4 and CD8 T cells remained negligible in TKO mice (Fig 3C and 3D). These data suggest that signaling through 2B4/SLAMF4 contributes to NKT cell development. To determine if SLAM, CD84, and Ly108 expression impacted NKT cell function, we measured *ex vivo* IL-4 and IFN-γ secretion after *in vivo* stimulation with α-GalCer. We found that WT NKT cells secreted significantly more cytokine compared to *Slamf1*,*5*,*6*^Δ/Δ^ NKT cells (IL-4, p = 0.0022; IFN-γ, p = 0.0152) (Fig 3E and 3F), suggesting that these SLAM family receptors supported cytokine secretion in NKT cells as they do in T cells [15,16].

### Absence of Tfh or germinal center defects in *Slamf1*,*5*,*6*^Δ/Δ^ mice

**Fig 4:**
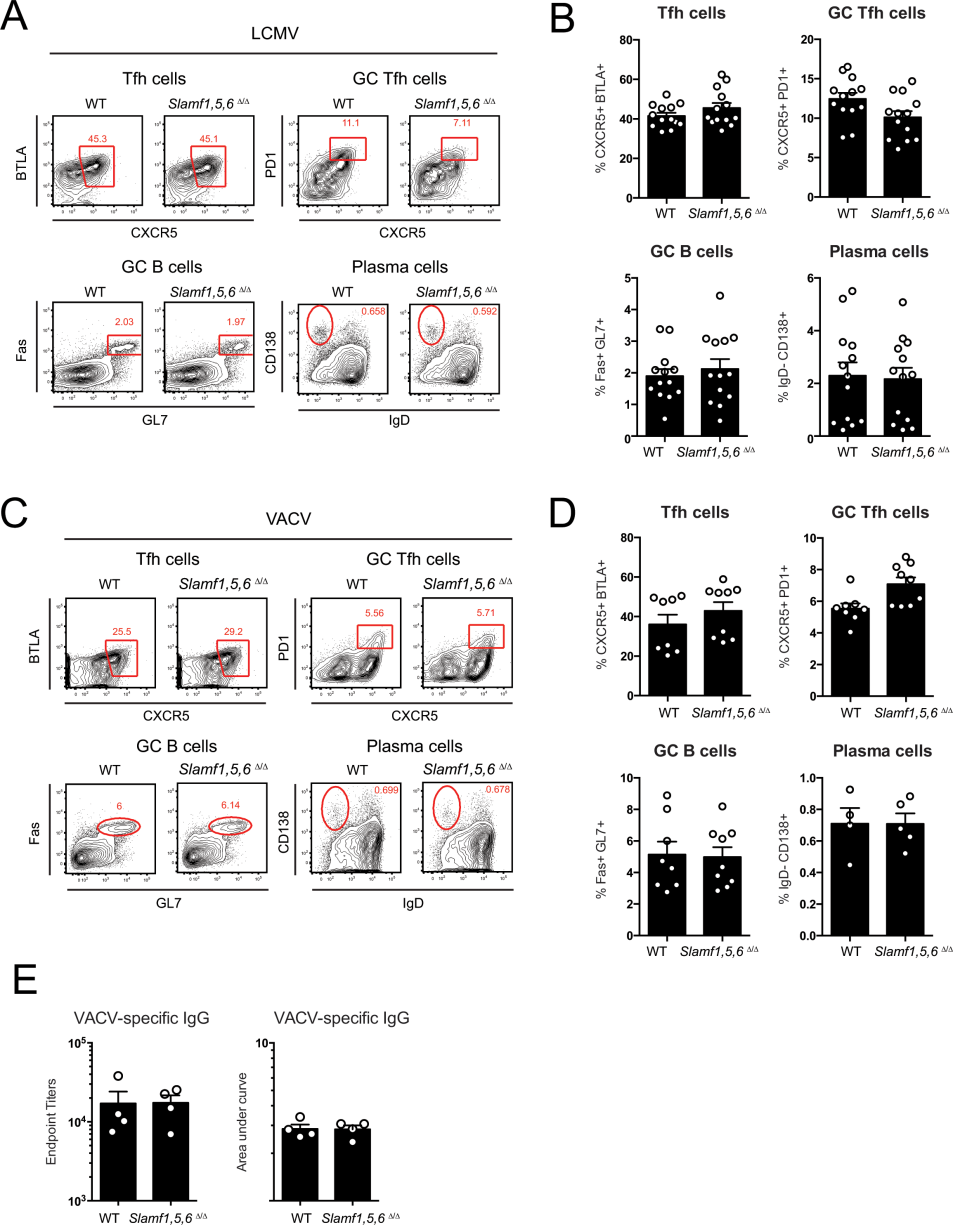
Absence of defects in germinal centers generated in *Slamf1*,*5*,*6* ^Δ/Δ^ mice after infection with LCMV and VACV. (A) Flow cytometry plots and (B) graphs of CD19^-^ CD4^+^ CD44^+^ CXCR5^+^ BTLA^+^ Tfh cells, CD19^-^ CD4^+^ CD44^+^ CXCR5^+^ PD1^+^ GC Tfh cells, CD4^-^ CD19^+^ Fas^+^ GL7^+^ GC B cells, and CD4^-^ CD19^+^ IgD^-^ CD138^+^ plasma cells in spleens of WT and *Slamf1*,*5*,*6* ^Δ/Δ^ mice at 8 days post infection with LCMV. (A-B) Data represents three independent experiments, with 4–5 mice per group. (C) Flow cytometry plots and (D) graphs of CD19^-^ CD4^+^ CD44^+^ CXCR5^+^ BTLA^+^ Tfh cells, CD19^-^ CD4^+^ CD44^+^ CXCR5^+^ PD1^+^ GC Tfh cells, CD4^-^ CD19^+^ Fas^+^ GL7^+^ GC B cells, and CD4^-^ CD19^+^ IgD^-^ CD138^+^ plasma cells in spleens of WT and *Slamf1*,*5*,*6* ^Δ/Δ^ mice at 8 days post infection with VACV. (C-D) Data represents two independent experiments, with 4 mice per group. (E) Endpoint titers and Area Under Curve (AUC) analyses of VACV specific serum IgG at 15 days post infection with VACV. Data represents one experiment, with four mice per group.

SAP expression in CD4 T cells is necessary for GC responses [7,35]. Because SAP binds to SLAM, CD84, and Ly108 on CD4 T cells, the impact of these receptors on GC responses was investigated. GC responses were measured in WT and *Slamf1*,*5*,*6*^Δ/Δ^ mice at 8 days post lymphocytic choriomeningitis virus (LCMV) infection. Frequencies of T follicular helper (Tfh) cells, GC Tfh cells, GC B cells and plasma cells (PCs) were comparable between WT and *Slamf1*,*5*,*6*^Δ/Δ^ mice (Fig 4A and 4B). To determine if this was a representative result, vaccinia virus (VACV) infection was used as a second acute viral infection model. Overall Tfh, GC Tfh, GC B cells, and plasma cell responses to VACV were comparable between WT and *Slamf1*,*5*,*6*^Δ/Δ^ mice (Fig 4C and 4D). We also found similar VACV-specific IgG titers in the serum of WT and *Slamf1*,*5*,*6*^Δ/Δ^ mice (Fig 4E), consistent with the comparable plasma cell responses seen in the spleen. This is in contrast to a report using *Slamf1*,*4*,*5*^-/-^ genomic locus deletion mice, where a slight increase in antibody responses was observed in the context of NP hapten immunization [22].

**Fig 5:**
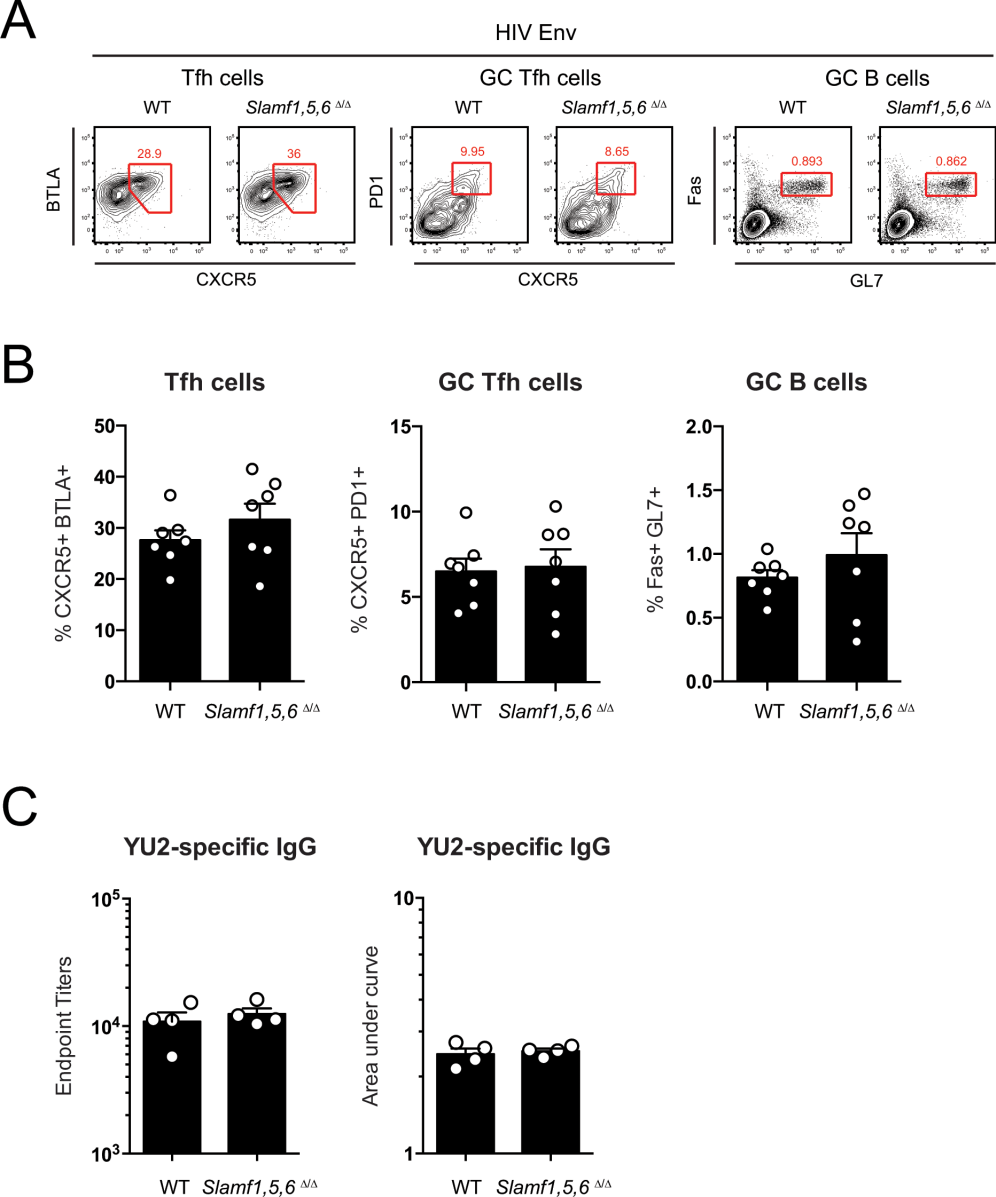
Lack of defects in germinal centers generated in *Slamf1*,*5*,*6* ^Δ/Δ^ mice after immunization with HIV envelope trimer protein. (A) Flow cytometry plots and (B) graphs of CD19^-^ CD4^+^ CD44^+^ CXCR5^+^ BTLA^+^ Tfh cells, CD19^-^ CD4^+^ CD44^+^ CXCR5^+^ PD1^+^ GC Tfh cells, and CD4^-^ CD19^+^ Fas^+^ GL7^+^ GC B cells in draining popliteal lymph nodes of of WT and *Slamf1*,*5*,*6* ^Δ/Δ^ mice at 8 days post immunization with HIV Envelope (YU2 gp140-F) protein. (A-B) Data represents two independent experiments, with 3–4 mice per grouop. (C) Endpoint titers and Area Under Curve (AUC) analyses of HIV Env (YU2-gp140-F) specific serum IgG at 15 days post immunization with YU2-gp140-F. Data represents one experiment, with 4 mice per group.

We considered the possibility that GC defects in *Slamf1*,*5*,*6*^Δ/Δ^ mice may be compensated by other pathways in the context of inflammatory acute viral infections, even though no compensation is seen in SAP-deficient mice. We therefore immunized mice with HIV envelope (Env) protein (YU2 gp140-F) in mild adjuvant as an independent model. Again, no differences were observed in Tfh, GC Tfh, or GC B cell responses between WT and *Slamf1*,*5*,*6*^Δ/Δ^ mice (Fig 5A and 5B). Additionally, there were no differences in HIV Env-specific IgG titers in serum (Fig 5C). Thus, in *Slamf1*,*5*,*6*^Δ/Δ^ mice, where all cells are deficient for SLAM, CD84, and Ly108 expression, no quantitative defects in Tfh or GC responses were observed in three independent models.

**Fig 6:**
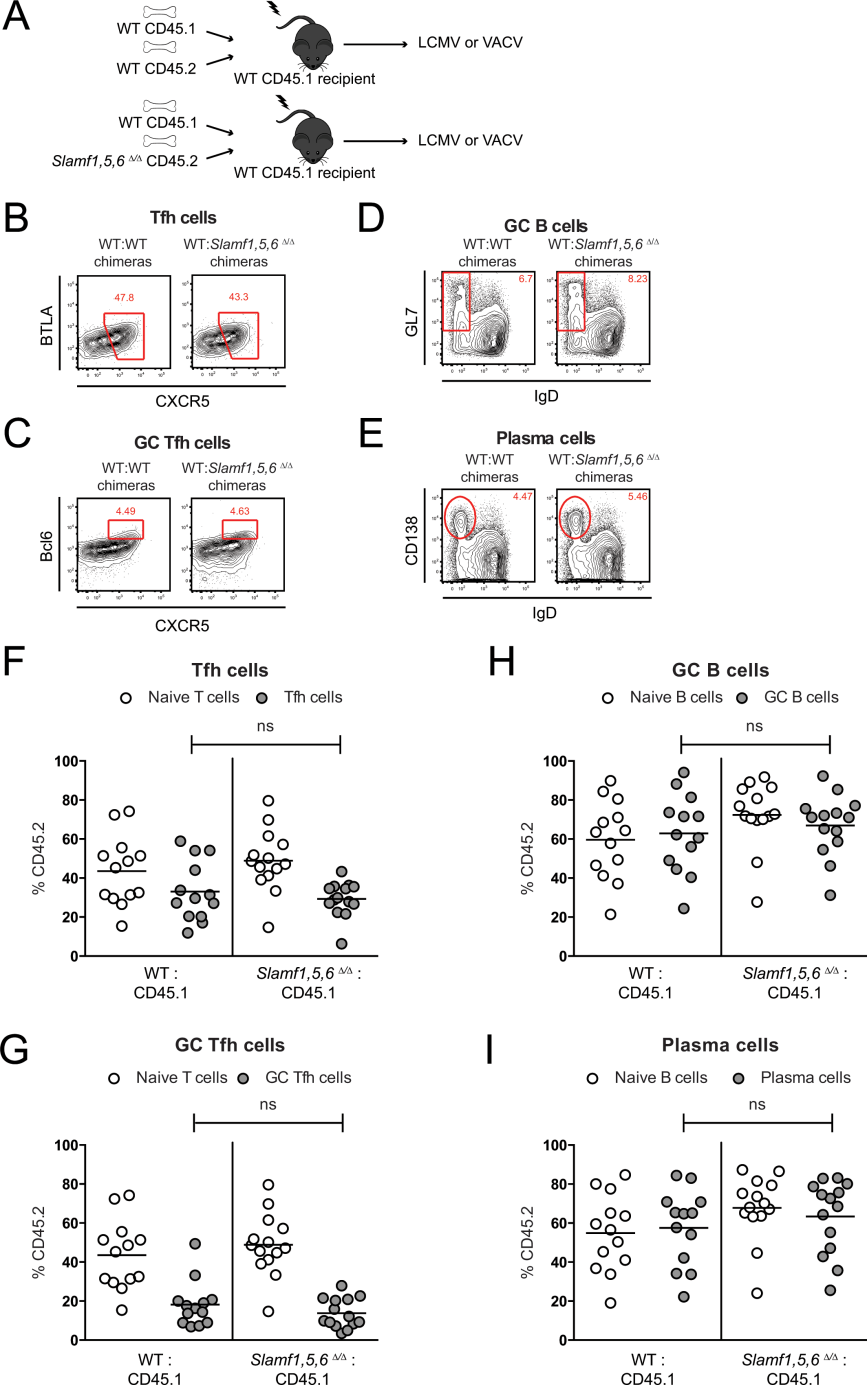
No competitive advantage for *Slamf1*, *Slamf5*, *and Slamf6*-expressing cells after LCMV infection. (A) Mixed WT CD45.1 and WT CD45.2 control bone marrow (BM) chimeras or mixed WT CD45.1 and SLAMf1,5,6 ^Δ/Δ^ CD45.2 BM chimeras were made and infected with LCMV or VACV. (B-I) Bone marrow chimeras infected with LCMV. Flow cytometry plots of CD19^-^ CD4^+^ CD44^+^ CXCR5^+^ BTLA^+^ Tfh cells (B), CD19^-^ CD4^+^ CD44^+^ CXCR5^+^ Bcl6^+^ GC Tfh cells (C), CD4^-^ CD19^+^ IgD^-^ GL7^+^ GC B cells (D) and CD4^-^ CD19^+^ IgD^-^ CD138^+^ plasma cells (E) in spleens of mixed WT:WT control BM chimeras or mixed WT:*SLAMf1*,*5*,*6* ^Δ/Δ^ BM chimeras at 7/8 days post infection with LCMV. (F-I) Frequencies of CD45.2^+^ WT or CD45.2^+^ *SLAMf1*,*5*,*6* ^Δ/Δ^ cells in WT:WT and WT:*SLAMf1*,*5*,*6* ^Δ/Δ^ BM chimeras at 7/8 days post LCMV infection. Tfh cells (F), GC Tfh cells (G), GC B cells (H), and plasma cells (I) are shown in relation to their naïve counterparts (naïve T cells or naïve B cells). (B-I) Data represent three independent experiments, with 4–5 mice per group.

**Fig 7:**
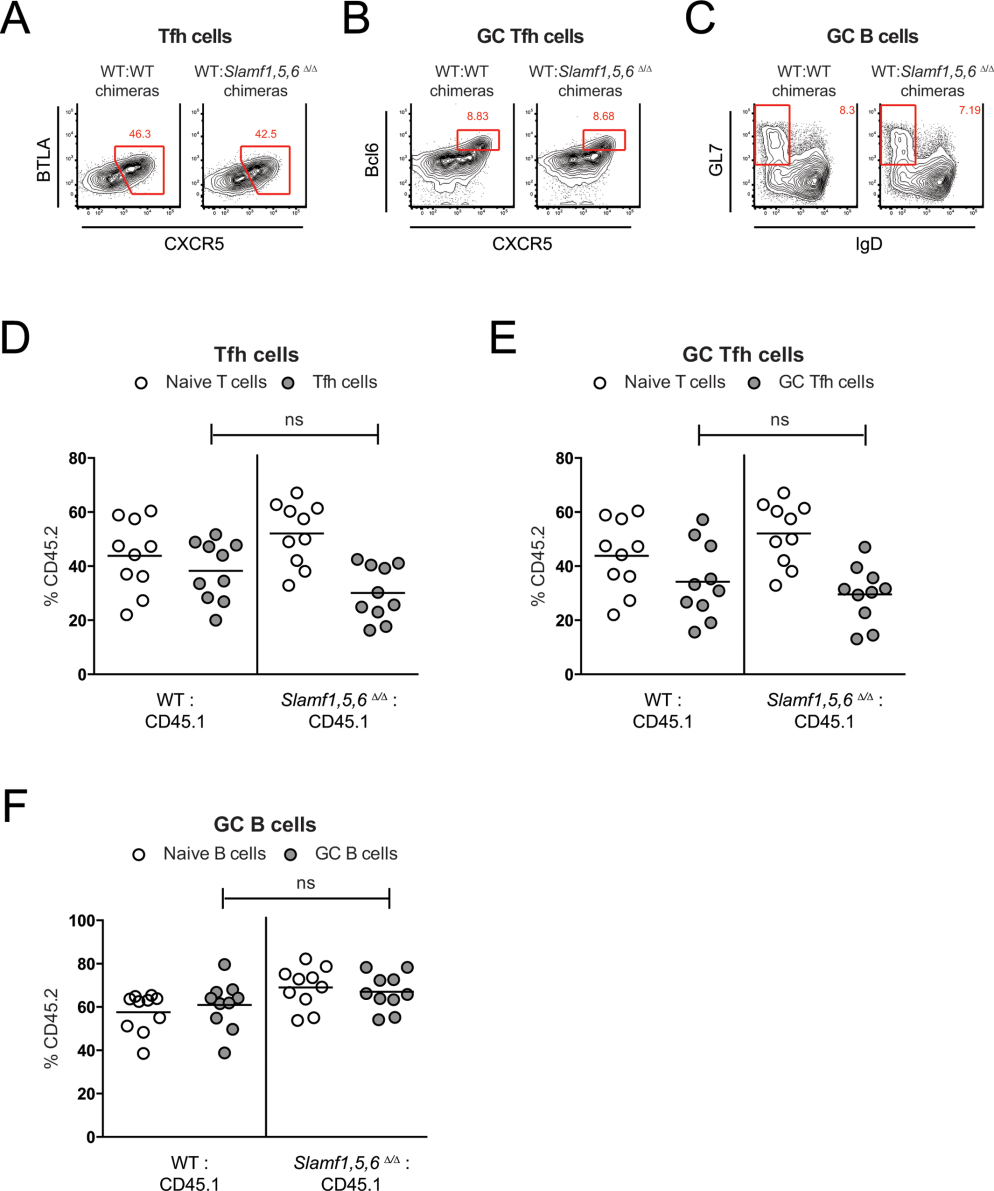
No competitive advantage for *Slamf1*, *Slamf5*, *and Slamf6*-expressing cells after VACV infection. (A-F) Bone marrow chimeras infected with VACV. Flow cytometry plots of CD19^-^ CD4^+^ CD44^+^ CXCR5^+^ BTLA^+^ Tfh cells (A), CD19^-^ CD4^+^ CD44^+^ CXCR5^+^ Bcl6^+^ GC Tfh cells (B), and CD4^-^ CD19^+^ IgD^-^ GL7^+^ GC B cells (C) in spleens of mixed WT:WT control BM chimeras or mixed WT:*SLAMf1*,*5*,*6* ^Δ/Δ^ BM chimeras at 7 days post infection with VACV. (D-F) Frequencies of CD45.2^+^ WT or CD45.2^+^ *SLAMf1*,*5*,*6* ^Δ/Δ^ cells in WT:WT and WT:*SLAMf1*,*5*,*6* ^Δ/Δ^ BM chimeras at 7 days post VACV infection. Tfh cells (D), GC Tfh cells (E), and GC B cells (F) are shown in relation to their naïve counterparts (naïve T cells or naïve B cells). (A-F) Data represent two independent experiments with 5 mice per group.

The roles of many factors can only be observed *in vivo* in the context of a competitive environment between WT and deficient cells. Therefore, bone marrow (BM) chimeras of B6.SJL (CD45.1^+/+^) reconstituted with WT (CD45.2^+/+^) and *Slamf1*,*5*,*6*^Δ/Δ^ CD45.2^+/+^ BM cells were tested for T and B cell responses after LCMV infection (Fig 6A). At 7/8 days post LCMV infection, Tfh cell, GC Tfh cell, GC B cell, and plasma cell responses were measured (Fig 6B–6E). There were no significant differences between the frequencies of WT and *Slamf1*,*5*,*6*^Δ/Δ^ cells (Fig 6F–6I). A second set of BM chimera experiments were performed with VACV infections. When Tfh cell, GC Tfh cell, and GC B cell responses were measured at 7 days post VACV infection (Fig 7A–7C), there were also no significant differences between the frequencies of WT and *Slamf1*,*5*,*6*^Δ/Δ^ cells (Fig 7D–7F). Thus, expression of SLAM, CD84, and Ly108 was not required for the initiation and maintenance of germinal center responses.

## Discussion

SLAMf receptors and SAP are required for NKT cell development, with the primary receptors thought to be SLAM and Ly108 [17,21]. In *Slamf1*,*5*,*6*^Δ/Δ^ mice, NKT cell development is defective, but not completely abolished as seen in SAP-deficient mice [32,33]. This partial defect in *Slamf1*,*5*,*6*^Δ/Δ^ mice, which confirms a recent report using *Slamf1*,*5*,*6*^*-/-*^ genomic locus deletion mice[21], suggests that other receptors are involved in NKT cell development. We then proceeded to further characterize the NKT cells that develop in *Slamf1*,*5*,*6*^Δ/Δ^ mice. We show a functional defect in NKT cells that develop and moreover, we find that 39% of splenic NKT cells in *Slamf1*,*5*,*6*^Δ/Δ^ mice aberrantly express 2B4/SLAMF4. We therefore conclude that 2B4 expression during development may largely compensate for loss of SLAM and Ly108 for NKT cell development, and that the 2B4 expression is then maintained in mature *Slamf1*,*5*,*6*^Δ/Δ^ NKT cells.

SLAMf receptors have been shown to play roles in CD4 T cell function, including cytokine secretion and sustained interactions with B cells. Thus, SLAMf receptors impact Tfh cell help to GC B cells, and in turn, GC B cells help maintain Tfh cells in GCs [2]. SAP is necessary for GC responses and long-term humoral immunity, and SAP expression in CD4 T cells aids in the recruitment and retention of GC Tfh cells within GCs [36]. For these reasons, we expected that multiple gene disruption in SLAM, CD84, and Ly108 would lead to defects in GCs. Therefore, the lack of overall defects in GC responses after viral infection and protein immunization even in the context of competition was unexpected. A recent report using *Slamf1*,*5*,*6*^*-/-*^ mice suggested that SLAM, CD84, and Ly108 negatively regulated antibody responses by recruiting SHP-1 to the B cell receptor (BCR) complex [22]. SHP-1 recruitment to the BCR is required for the maintenance of GCs [37], but it is not known how SLAM family receptors are involved in this regulation. Wang et al. show an approximately 2-fold increase in antigen specific IgG titers and plasma cell frequencies in *Slamf1*,*5*,*6*^*-/-*^ mice. In contrast, we observed no enhancement of plasma cell or Ab responses in the *Slamf1*,*5*,*6*^Δ/Δ^ mice in three independent systems. Furthermore, Wang et al. show enhanced antibody titers from *Slamf1*,*5*,*6*^*-/-*^ B cells transferred into *Rag-1*^-/-^ mice whereas we show similar plasma cell responses in WT and *Slamf1*,*5*,*6*^Δ/Δ^ bone marrow chimeras. Differences in these results may relate to differences in experimental systems, with some systems more capable of revealing subtle differences than others. Yet, the use of alternatively generated SLAMf receptor deficient mice and different experimental systems is beneficial to our understanding of the roles of these complex receptors in the immune response. Both Terhorst and colleagues and our group found no overt differences in Tfh cells and GC B cell responses. This lack of phenotype in *Slamf1*,*5*,*6*^Δ/Δ^ mice did not correspond with the severe GC defects seen SAP^-/-^ mice. This suggests that the SAP^-/-^ defect is almost exclusively caused by potent negative signaling via Ly108 in the absence of SAP [12]. Our data also suggests that SLAM, CD84, and Ly108 have functionally redundant roles with other receptors involved in GC responses. Additional receptor mediated interactions impact the recruitment and maintenance of GC Tfh cells, such as chemokine receptors, integrins, and ICOS:ICOSL interactions between Tfh cells and B cells [38]. Thus, SLAMf receptor expression impacts GC responses likely by enhancing T:B cell interactions. Meanwhile, other receptors may perform partially redundant functions in T:B interactions that help orchestrate the complex interactions occurring in GCs. Lastly, because SLAMf receptors have the potential to signal both positively and negatively through immunotyrosine switch motifs (ITSMs) [5], the interpretation of these SLAMf receptor TKO phenotypes is complicated by impacts on functions downstream of both positive and negative signaling. Further exploration using multiple SLAMf receptor deficient mice to understand these phenotypes and the mechanisms underlying these phenotypes would be worthwhile.

## Supporting Information

S1 Fig Comparison of WT vs. *Slamf1*,*5*,*6*^Δ/Δ^ breeder lines after LCMV infection. WT vs. *Slamf1*,*5*,*6*^Δ/Δ^ GC B cells, plasma cells, and GC Tfh cells at 8 days post-LCMV infection. Analysis of variance (ANOVA) comparing WT vs. *Slamf1*,*5*,*6*^Δ/Δ^ breeder lines show no significant differences.
(EPS)

S2 Fig Expression of CD84 on CD4 T cells and B cells in *Slamf1*,*5*,*6*^Δ/Δ^ mice. CD84 expression on CD4 T cells and B cells from mouse #5 and mouse #6 (Table 2), which were confirmed genotypically as *Slamf1*,*5*,*6*^Δ/Δ^ mice. CD84 expression on CD4 T cells and B cells from WT mice (blue) are shown as a comparison to CD84 expression in *Slamf1*,*5*,*6*^Δ/Δ^ mice (dashed gray).
(EPS)
